# Supplementary material for: A model of multiple tumor marker for lymph node metastasis assessment in colorectal cancer: a retrospective study
Source: PeerJ. 2022 Apr 11;10:e13196. doi: 10.7717/peerj.13196 (PMC9009328; doi:10.7717/peerj.13196)
Supplement: Supplemental Information 1 [file peerj-10-13196-s001.docx]

| **Table S1 Inclusion and exclusion criteria** | |
| --- | --- |
| Inclusion criteria | Exclusion criteria |
| Patients who underwent surgery for CRC with curative intent | Patients who had received chemotherapy or radiotherapy for other malignant tumors |
| Preoperative biopsy-proven histological grade available. | Patients received chemotherapy or radiotherapy in other hospitals before surgery |
| Patient's data of tumor marker is complete | Patients with non-primary CRC |

Abbreviations: CRC, colorectal cancer.

**Table S2. Cutoff value of tumor markers.**

| Item | Cutoff value |
| --- | --- |
| AFP | 0.01 |
| CEA | -0.168 |
| CA125 | -0.066 |
| CA153 | 0.61 |
| CA19-9 | -0.17 |

Abbreviations: AFP, alpha fetoprotein; CEA, carcinoembryonic antigen; CA125, carbohydrate antigen 125; CA153, carbohydrate antigen 153; CA19-9, carbohydrate antigen 19-9.

| **Table S3. Characteristics difference between primary, internal test, and external test cohorts** | | | | |
| --- | --- | --- | --- | --- |
| Characteristic | Primary cohort | Internal test cohort | External test cohort | *P* |
| Age, mean ± SD, years | 61.70 ± 11.33 | 60.27 ± 11.68 | 63.56 ± 13.39 | 0.030* |
| Sex, No. (%) |  |  |  | 0.247 |
| Male | 290 (61.57) | 119 (58.62) | 119 (66.85) |  |
| Female | 181 (38.43) | 84 (41.38) | 59 (33.15) |  |
| T stage, No. (%) |  |  |  | 0.299 |
| 1 | 136 (28.87) | 54 (26.60) | 41 (23.04) |  |
| 2 | 256 (54.35) | 117 (57.64) | 97 (54.49) |  |
| 3 | 79 (16.77) | 32 (15.76) | 40 (22.47) |  |
| CEA level, No. (%) |  |  |  | 0.031* |
| Normal | 276 (58.60) | 117 (57.64) | 123 (69.10) |  |
| Abnormal | 195 (41.40) | 86 (42.36) | 55 (30.90) |  |
| AFP level, No. (%) |  |  |  | 0.007* |
| Normal | 432 (91.72) | 185 (91.13) | 175 (98.31) |  |
| Abnormal | 39 (8.28) | 18 (8.87) | 3 (1.69) |  |
| CA125 level, No. (%) |  |  |  | 0.485 |
| Normal | 332 (70.49) | 147 (72.41) | 119 (66.85) |  |
| Abnormal | 139 (29.51) | 56 (27.59) | 59 (33.15) |  |
| CA153 level, No. (%) |  |  |  | 0.005* |
| Normal | 385 (81.74) | 159 (78.32) | 161 (90.45) |  |
| Abnormal | 86 (18.26) | 44 (21.67) | 17 (9.55) |  |
| CA19-9 level, No. (%) |  |  |  | 0.413 |
| Normal | 291 (61.78) | 128 (63.05) | 120 (67.42) |  |
| Abnormal | 180 (38.22) | 75 (36.95) | 58 (32.58) |  |
| LNM |  |  |  | 0.736 |
| - | 275 (58.39) | 118 (58.13) | 98 (55.06) |  |
| + | 196 (41.61) | 85 (41.87) | 80 (44.94) |  |

*NOTE*. *P* value is derived from the univariable association analyses between each of the clinicopathologic variables and metastasis status. The T stage 1 represents pathological stage T1 and T2, T stage 1e 2 represents pathological stage T3 and T stage 1 represents pathological stage T4.

Abbreviations: CEA, carcinoembryonic antigen; AFP, alpha fetoprotein; CA125, carbohydrate antigen 125; CA153, carbohydrate antigen 153; CA19-9, carbohydrate antigen 19-9; LNM, lymph node metastasis; SD, standard deviation.

**P* value < 0.05.

| **Table S4. Characteristics difference between primary, internal test, and external test cohorts in the metastasis prediction model** | | | | |
| --- | --- | --- | --- | --- |
| Characteristic | Primary cohort | Internal test cohort | External test cohort | *P* |
| Age, mean ± SD, years | 61.52 ± 11.40 | 60.68 ± 11.58 | 63.56 ± 13.39 | 0.048* |
| Sex, No. (%) |  |  |  | 0.148 |
| Male | 293 (62.21) | 116 (57.14) | 119 (66.85) |  |
| Female | 178 (37.79) | 87 (42.86) | 59 (33.15) |  |
| T stage, No. (%) |  |  |  | 0.179 |
| 1 | 127 (26.96) | 63 (31.03) | 41 (23.04) |  |
| 2 | 269 (57.11) | 104 (51.23) | 97 (54.49) |  |
| 3 | 75 (15.93) | 36 (17.74) | 40 (22.47) |  |
| CEA level, No. (%) |  |  |  | 0.005* |
| Normal | 286 (60.72) | 107 (52.71) | 123 (69.10) |  |
| Abnormal | 185 (39.28) | 96 (47.29) | 55 (30.90) |  |
| AFP level, No. (%) |  |  |  | 0.006* |
| Normal | 429 (91.08) | 188 (92.61) | 175 (98.31) |  |
| Abnormal | 42 (8.92) | 15 (7.39) | 3 (1.69) |  |
| CA125 level, No. (%) |  |  |  | 0.226 |
| Normal | 342 (72.61) | 137 (67.49) | 119 (66.85) |  |
| Abnormal | 129 (27.39) | 66 (32.51) | 59 (33.15) |  |
| CA153 level, No. (%) |  |  |  | 0.001* |
| Normal | 389 (82.59) | 155 (76.35) | 161 (90.45) |  |
| Abnormal | 82 (17.41) | 48 (23.65) | 17 (9.55) |  |
| CA19-9 level, No. (%) |  |  |  | 0.157 |
| Normal | 301 (63.91) | 118 (58.13) | 120 (67.42) |  |
| Abnormal | 170 (36.09) | 85 (41.87) | 58 (32.58) |  |
| Metastasis |  |  |  | 0.326 |
| - | 268 (56.90) | 115 (56.65) | 90 (50.56) |  |
| + | 203 (43.10) | 88 (43.35) | 88 (49.44) |  |

*NOTE*. *P* value is derived from the univariable association analyses between each of the clinicopathologic variables and metastasis status. The T stage 1 represents pathological stage T1 and T2, T stage 1e 2 represents pathological stage T3 and T stage 1 represents pathological stage T4.

Abbreviations: CEA, carcinoembryonic antigen; AFP, alpha fetoprotein; CA125, carbohydrate antigen 125; CA153, carbohydrate antigen 153; CA19-9, carbohydrate antigen 19-9; SD, standard deviation.

**P* value < 0.05.

| **Table S5. Relevant results of important risk factors** | | | | | | |
| --- | --- | --- | --- | --- | --- | --- |
| Item | Specificity (95%CI) | Sensitivity (95%CI) | Accuracy (95%CI) | PPV (95%CI) | NPV (95%CI) | AUC (95%CI) |
| AFP | 0.629 (0.282,0.786) | 0.488 (0.309,0.808) | 0.567 (0.507,0.602) | 0.498 (0.454,0.552) | 0.617 (0.586,0.662) | 0.534 (0.490,0.578) |
| CEA | 0.699 (0.415,0.893) | 0.533 (0.309,0.801) | 0.629 (0.574,0.665) | 0.581 (0.504,0.701) | 0.660 (0.624,0.740) | 0.635 (0.593,0.678) |
| CA125 | 0.747 (0.457,0.940) | 0.361 (0.127,0.653) | 0.579 (0.528,0.611) | 0.516 (0.464,0.621) | 0.607 (0.583,0.644) | 0.534 (0.489,0.578) |
| CA153 | 0.634 (0.034,0.982) | 0.433 (0.048,0.993) | 0.555 (0.445,0.591) | 0.476 (0.436,0.661) | 0.592 (0.574,0.875) | 0.490 (0.446,0.535) |
| CA19-9 | 0.744 (0.574,0.869) | 0.533 (0.385,0.694) | 0.651 (0.611,0.687) | 0.614 (0.542,0.701) | 0.676 (0.641,0.719) | 0.649 (0.606,0.692) |
| Cutoff_AFP | 0.736 (0.689,0.778) | 0.529 (0.474,0.584) | 0.647 (0.611,0.680) | 0.604 (0.556,0.649) | 0.673 (0.643,0.702) | 0.633 (0.597,0.669) |
| Cutoff_CEA | 0.671 (0.627,0.718) | 0.533 (0.474,0.591) | 0.611 (0.574,0.648) | 0.552 (0.507,0.597) | 0.654 (0.621,0.687) | 0.602 (0.565,0.639) |
| Cutoff_CA125 | 0.752 (0.710,0.794) | 0.340 (0.289,0.399) | 0.576 (0.540,0.608) | 0.512 (0.453,0.573) | 0.601 (0.577,0.625) | 0.548 (0.513,0.583) |
| Cutoff_CA153 | 0.822 (0.789,0.859) | 0.213 (0.175,0.261) | 0.559 (0.542,0.588) | 0.477 (0.434,0.551) | 0.579 (0.568,0.596) | 0.515 (0.484,0.545) |
| Cutoff_CA19-9 | 0.736 (0.695,0.781) | 0.529 (0.471,0.591) | 0.647 (0.613,0.682) | 0.604 (0.559,0.653) | 0.673 (0.645,0.705) | 0.633 (0.597,0.669) |
| CA199/AFP | 0.663 (0.509,0.880) | 0.615 (0.364,0.756) | 0.639 (0.601,0.677) | 0.579 (0.529,0.700) | 0.694 (0.645,0.745) | 0.646(0.604,0.689) |
| CA125/AFP | 0.747 (0.321,0.927) | 0.371 (0.148,0.763) | 0.582 (0.507,0.613) | 0.522 (0.457,0.6222) | 0.609 (0.585,0.653) | 0.540(0.496,0.584) |
| CA153/AFP | 0.717 (0.520,0.958) | 0.378 (0.093,0.570) | 0.570 (0.525,0.604) | 0.502 (0.453,0.633) | 0.596 (0.577,0.633) | 0.509(0.464,0.554) |
| CA153/CA125 | 0.726 (0.175,0.966) | 0.376 (0.093,0.900) | 0.576 (0.488,0.610) | 0.515 (0.451,0.651) | 0.604 (0.582,0.692) | 0.534(0.490,0.579) |
| CA199/CA125 | 0.752 (0.648,0.898) | 0.495 (0.316,0.598) | 0.639 (0.604,0.672) | 0.601 (0.544,0.720) | 0.660 (0.628,0.694) | 0.620(0.577,0.664) |
| CA199/CA153 | 0.715 (0.629,0.906) | 0.546 (0.320,0.643) | 0.645 (0.610,0.678) | 0.598 (0.547,0.731) | 0.673 (0.634,0.711) | 0.644(0.601,0.686) |
| CEA/AFP | 0.593 (0.441,0.809) | 0.639 (0.392,0.784) | 0.610 (0.567,0.650) | 0.540 (0.499,0.632) | 0.682 (0.628,0.747) | 0.630(0.587,0.672 |
| CEA/CA125 | 0.734 (0.600,0.810) | 0.526 (0.430,0.639) | 0.642 (0.605,0.678) | 0.598 (0.544,0.654) | 0.670 (0.638,0.705) | 0.614(0.570,0.657) |
| CEA/CA153 | 0.731 (0.590,0.812) | 0.526 (0.430,0.660) | 0.641 (0.605,0.675) | 0.596 (0.542,0.653) | 0.670 (0.639,0.704) | 0.635(0.593,0.678) |
| CEA/CA199 | 0.890 (0.987,0.611) | 0.234 (0.103,0509) | 0.605 (0.571,0.629) | 0.616 (0.505,0.842) | 0.605 (0.588,0.625) | 0.527(0.482,0.572) |

| **Table S6. Performance of the metastasis prediction models** | | | | |
| --- | --- | --- | --- | --- |
|  | Internal test cohort | | External test cohort | |
|  | 1se model | Min model | 1se model | Min model |
| Specificity (95%CI) | 0.800 (0.330,0.887) | 0.704 (0.469,0.922) | 0.789 (0.356,0.933) | 0.800 (0.522,0.900) |
| Sensitivity (95%CI) | 0.568 (0.455,0.966) | 0.727 (0.477,0.920) | 0.545 (0.352,0.920) | 0.648 (0.523,0.886) |
| Accuracy (95%CI) | 0.695 (0.601,0.754) | 0.709 (0.640,0.768) | 0.657 (0.601,0.725) | 0.719 (0.657,0.787) |
| PPV (95%CI) | 0.683 (0.522,0.792) | 0.652 (0.555,0.827) | 0.708 (0.574,0.842) | 0.756 (0.637,0.851) |
| NPV (95%CI) | 0.710 (0.662,0.940) | 0.773 (0.688,0.900) | 0.636 (0.580,0.825) | 0.698 0.637,0.8222) |
| AUC (95%CI) | 0.724 (0.656,0.791) | 0.760 (0.694,0.826) | 0.704 (0.631,0.777) | 0.740 (0.667,0.814) |

Abbreviations: Min, minimum criteria; 1se, 1 standard error of the minimum criteria.
