# Supplementary material for: A model of multiple tumor marker for lymph node metastasis assessment in colorectal cancer: a retrospective study
Source: PeerJ. 2022 Apr 11;10:e13196. doi: 10.7717/peerj.13196 (PMC9009328; doi:10.7717/peerj.13196)
Supplement: Supplemental Information 2 [file peerj-10-13196-s002.ppt]

## Slide 1
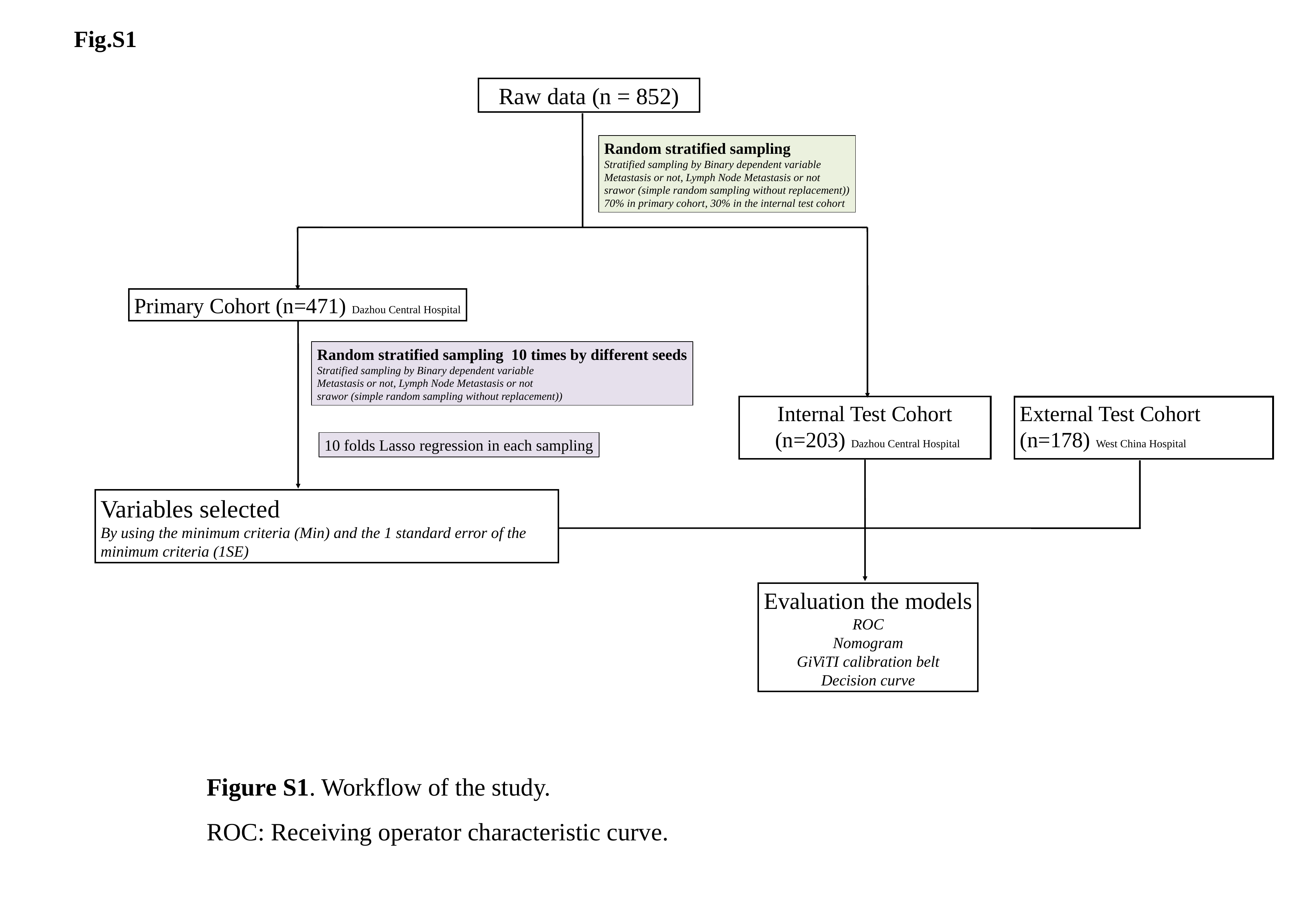

Fig.S1
Raw data (n = 852)
Random stratified sampling
Stratified sampling by Binary dependent variable
Metastasis or not, Lymph Node Metastasis or not
srawor (simple random sampling without replacement))
70% in primary cohort, 30% in the internal test cohort
Primary Cohort (n=471) Dazhou Central Hospital
Random stratified sampling 10 times by different seeds
Stratified sampling by Binary dependent variable
Metastasis or not, Lymph Node Metastasis or not
srawor (simple random sampling without replacement))
Internal Test Cohort
 (n=203) Dazhou Central Hospital
External Test Cohort
(n=178) West China Hospital
10 folds Lasso regression in each sampling
Variables selected
By using the minimum criteria (Min) and the 1 standard error of the minimum criteria (1SE)
Evaluation the models
ROC
Nomogram
GiViTI calibration belt
Decision curve
Figure S1. Workflow of the study.
ROC: Receiving operator characteristic curve.

## Slide 2
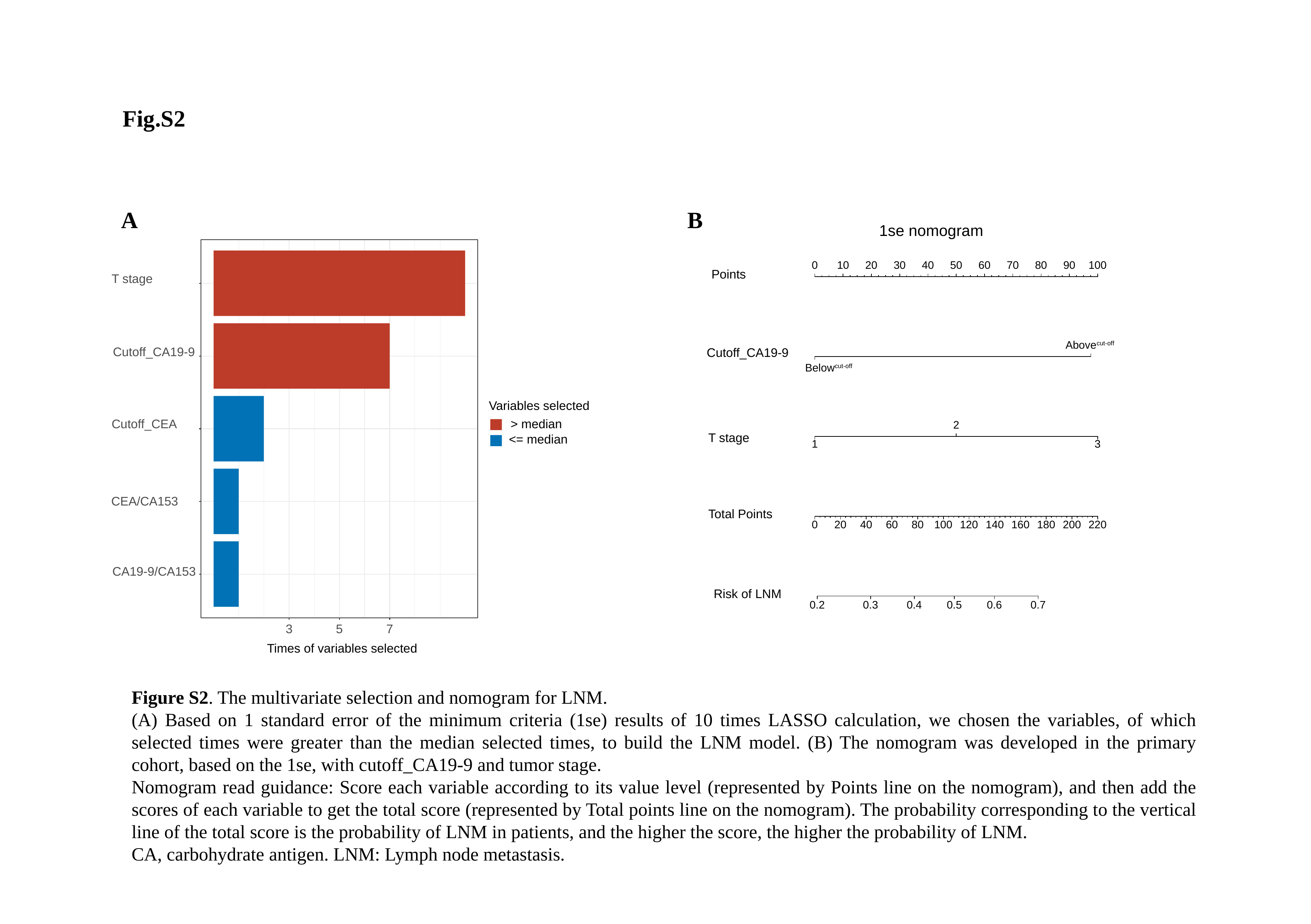

Fig.S2
A
 T stage
 Cutoff_CA19-9
Variables selected
 Cutoff_CEA
> median
<= median
CEA/CA153
 CA19-9/CA153
3
5
7
Times of variables selected
B
1se nomogram
0
10
20
30
40
50
60
70
80
90
100
Points
 Cutoff_CA19-9
Belowcut-off
2
 T stage
1
3
Total Points
0
20
40
60
80
100
120
140
160
180
200
220
Risk of LNM
0.2
0.3
0.4
0.5
0.6
0.7
Abovecut-off
Figure S2. The multivariate selection and nomogram for LNM.
(A) Based on 1 standard error of the minimum criteria (1se) results of 10 times LASSO calculation, we chosen the variables, of which selected times were greater than the median selected times, to build the LNM model. (B) The nomogram was developed in the primary cohort, based on the 1se, with cutoff_CA19-9 and tumor stage.
Nomogram read guidance: Score each variable according to its value level (represented by Points line on the nomogram), and then add the scores of each variable to get the total score (represented by Total points line on the nomogram). The probability corresponding to the vertical line of the total score is the probability of LNM in patients, and the higher the score, the higher the probability of LNM.
CA, carbohydrate antigen. LNM: Lymph node metastasis.

## Slide 3
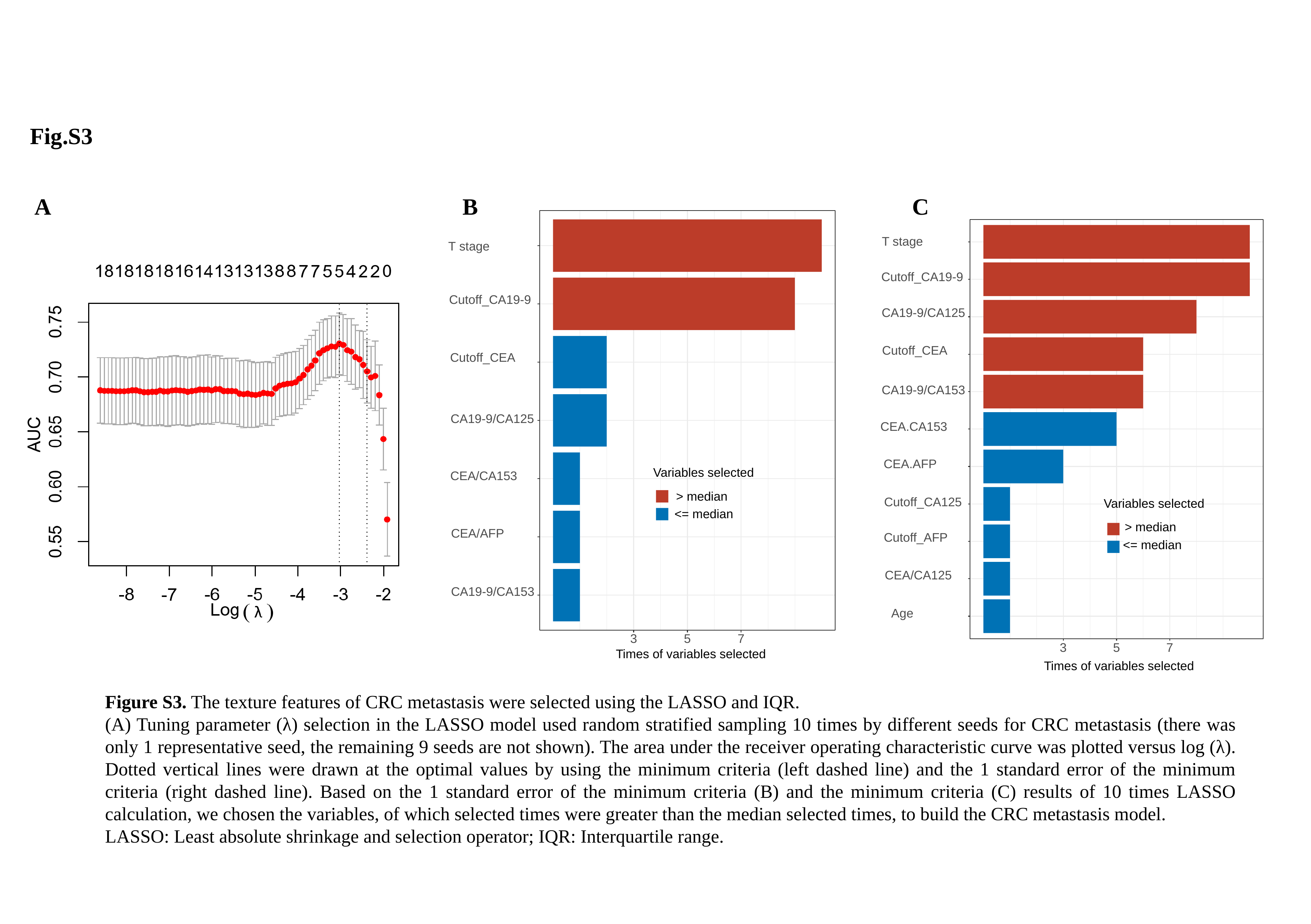

Fig.S3
A
B
C
 Cutoff_CA19-9
 Cutoff_CEA
 CA19-9/CA125
Variables selected
 CEA/CA153
> median
<= median
 CEA/AFP
 CA19-9/CA153
3
5
7
Times of variables selected
T stage
 T stage
 Cutoff_CA19-9
 CA19-9/CA125
 Cutoff_CEA
 CA19-9/CA153
CEA.CA153
CEA.AFP
 Cutoff_CA125
Variables selected
> median
 Cutoff_AFP
<= median
CEA/CA125
Age
3
5
7
Times of variables selected
Figure S3. The texture features of CRC metastasis were selected using the LASSO and IQR.
(A) Tuning parameter (λ) selection in the LASSO model used random stratified sampling 10 times by different seeds for CRC metastasis (there was only 1 representative seed, the remaining 9 seeds are not shown). The area under the receiver operating characteristic curve was plotted versus log (λ). Dotted vertical lines were drawn at the optimal values by using the minimum criteria (left dashed line) and the 1 standard error of the minimum criteria (right dashed line). Based on the 1 standard error of the minimum criteria (B) and the minimum criteria (C) results of 10 times LASSO calculation, we chosen the variables, of which selected times were greater than the median selected times, to build the CRC metastasis model.
LASSO: Least absolute shrinkage and selection operator; IQR: Interquartile range.

## Slide 4
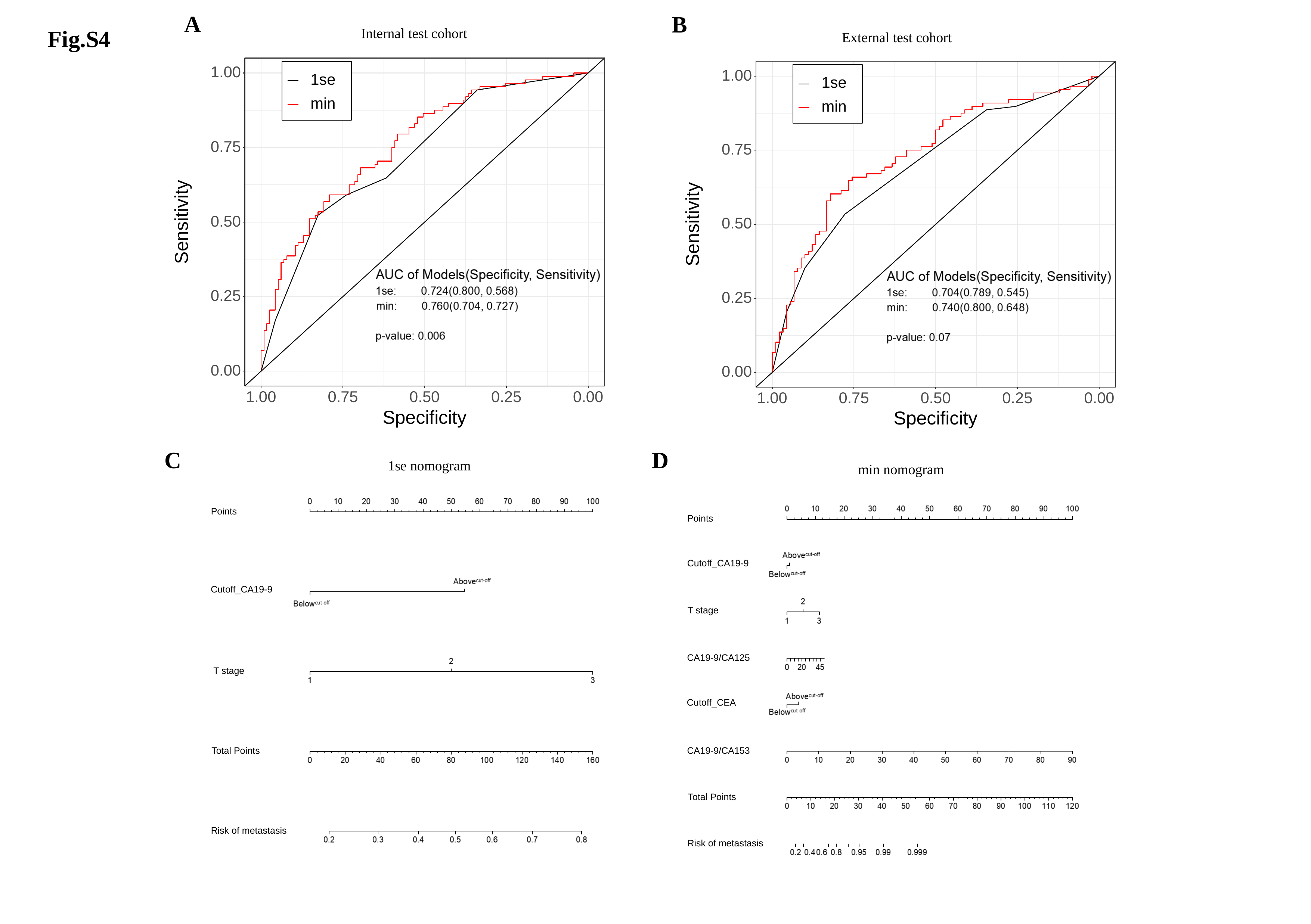

A
B
Fig.S4
Internal test cohort
External test cohort
1.00
1.00
1se
1se
min
min
0.75
0.75
Sensitivity
Sensitivity
0.50
0.50
0.25
0.25
0.00
0.00
1.00
0.75
0.50
0.25
0.00
1.00
0.75
0.50
0.25
0.00
Specificity
Specificity
C
D
Points
 Cutoff_CA19-9
 T stage
Total Points
Risk of metastasis
1se nomogram
min nomogram
Points
 Cutoff_CA19-9
 T stage
 CA19-9/CA125
 Cutoff_CEA
 CA19-9/CA153
Total Points
Risk of metastasis

## Slide 5
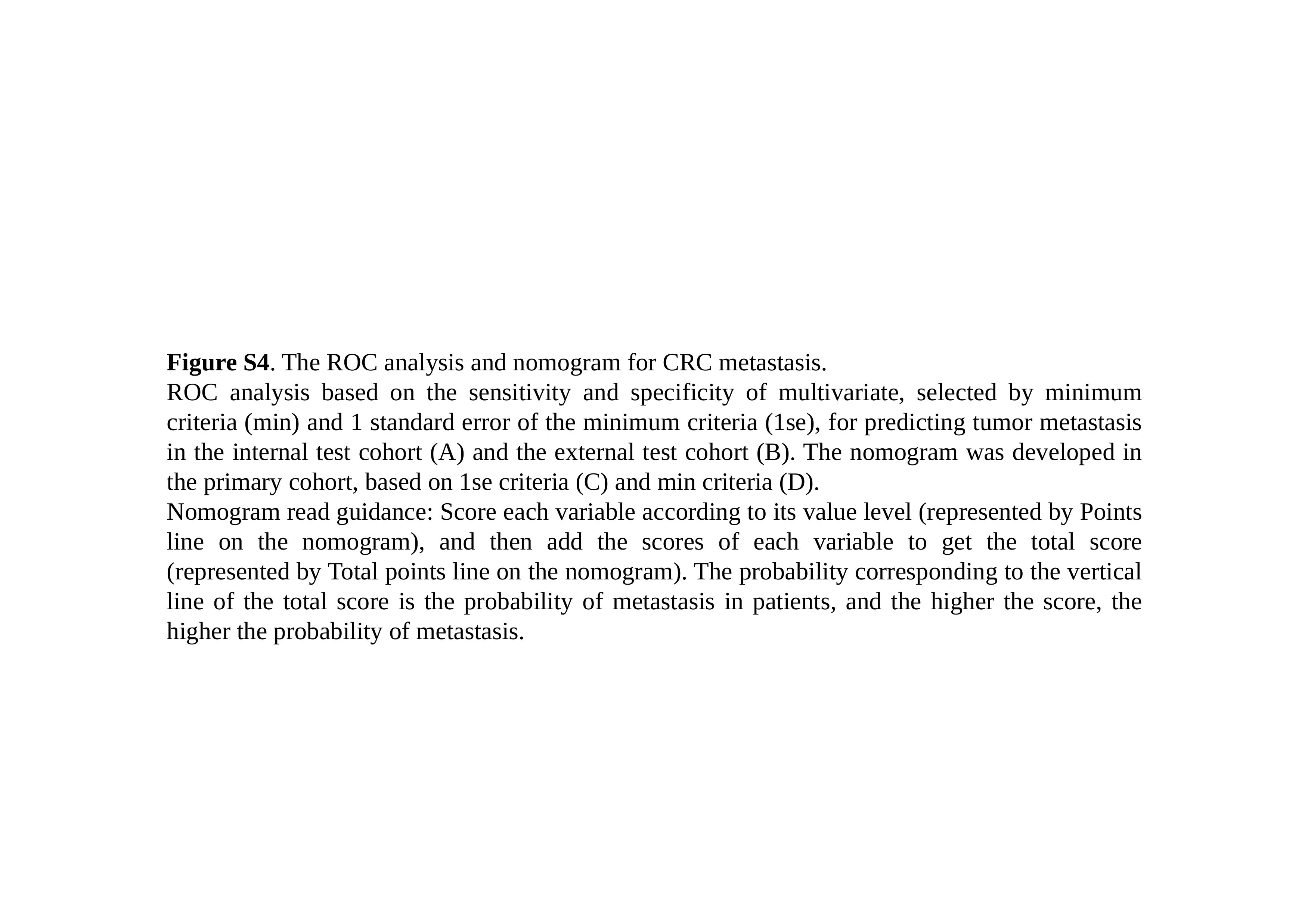

Figure S4. The ROC analysis and nomogram for CRC metastasis.
ROC analysis based on the sensitivity and specificity of multivariate, selected by minimum criteria (min) and 1 standard error of the minimum criteria (1se), for predicting tumor metastasis in the internal test cohort (A) and the external test cohort (B). The nomogram was developed in the primary cohort, based on 1se criteria (C) and min criteria (D).
Nomogram read guidance: Score each variable according to its value level (represented by Points line on the nomogram), and then add the scores of each variable to get the total score (represented by Total points line on the nomogram). The probability corresponding to the vertical line of the total score is the probability of metastasis in patients, and the higher the score, the higher the probability of metastasis.

## Slide 6
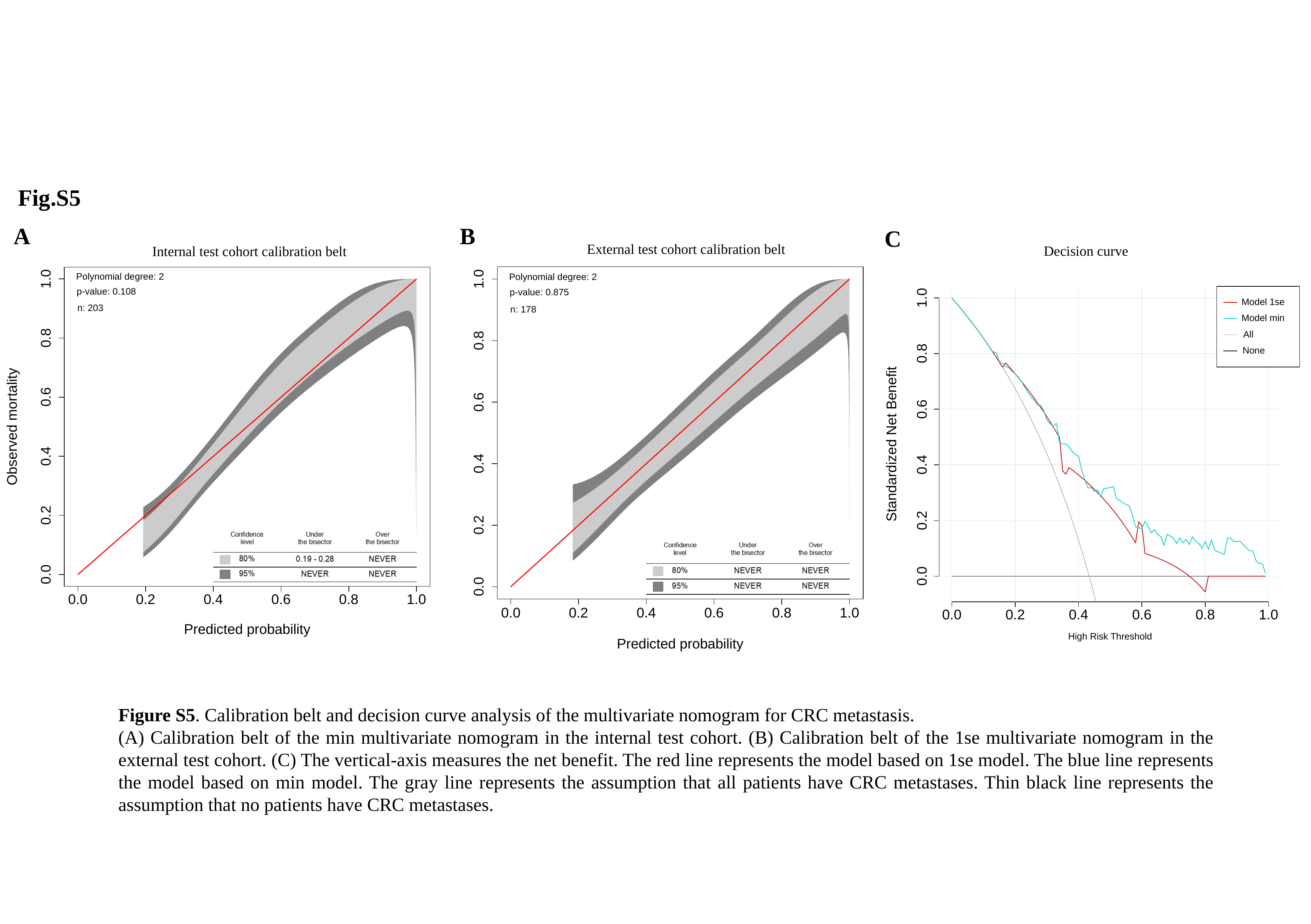

Fig.S5
A
B
C
Polynomial degree: 2
1.0
p-value: 0.875
n: 178
0.8
0.6
Observed mortality
0.4
0.2
0.0
0.0
0.2
0.4
0.6
0.8
1.0
Predicted probability
Polynomial degree: 2
1.0
p-value: 0.108
n: 203
0.8
0.6
Observed mortality
0.4
0.2
0.0
0.0
0.2
0.4
0.6
0.8
1.0
Predicted probability
External test cohort calibration belt
Decision curve
Internal test cohort calibration belt
1.0
Model 1se
Model min
All
None
0.8
0.6
Standardized Net Benefit
0.4
0.2
0.0
0.0
0.2
0.4
0.6
0.8
1.0
High Risk Threshold
Figure S5. Calibration belt and decision curve analysis of the multivariate nomogram for CRC metastasis.
(A) Calibration belt of the min multivariate nomogram in the internal test cohort. (B) Calibration belt of the 1se multivariate nomogram in the external test cohort. (C) The vertical-axis measures the net benefit. The red line represents the model based on 1se model. The blue line represents the model based on min model. The gray line represents the assumption that all patients have CRC metastases. Thin black line represents the assumption that no patients have CRC metastases.
